# Efficacy of attractive targeted sugar bait stations against malaria in Western Province Zambia: epidemiological findings from a two-arm cluster randomized phase III trial

**DOI:** 10.1186/s12936-024-05175-8

**Published:** 2024-11-15

**Authors:** Ruth A. Ashton, Kochelani Saili, Chama Chishya, Handrinah Banda Yikona, Annie Arnzen, Erica Orange, Chanda Chitoshi, John Chulu, Titus Tobolo, Frank Ndalama, Irene Kyomuhangi, Willy Ngulube, Hawela Moonga, Jacob Chirwa, Laurence Slutsker, Joseph Wagman, Javan Chanda, John Miller, Kafula Silumbe, Busiku Hamainza, Thomas P. Eisele, Joshua Yukich, Megan Littrell

**Affiliations:** 1 Centre for Applied Malaria Research and Evaluation, Tulane School of Public Health and Tropical Medicine, 1440 Canal Street, New Orleans, LA 70112 USA; 2 PATH, Kaoma, Zambia; 3 PATH, Seattle, WA USA; 4 Centre for Health Informatics, Computing, and Statistics, Lancaster University, Lancaster, UK; 5 National Malaria Elimination Centre, Lusaka, Zambia; 6 Independent Consultant, Atlanta, GA USA; 7 PATH, Washington, DC USA; 8 PATH, Lusaka, Zambia; 9 Present Address: Macha Research Trust, Choma, Zambia

**Keywords:** Attractive targeted sugar bait, Vector control, Zambia, Cluster randomized controlled trial

## Abstract

**Background:**

Attractive targeted sugar bait (ATSB) stations containing bait (to attract) and ingestion toxicant (to kill) sugar-foraging mosquitoes are hypothesized to reduce malaria transmission by shortening the lifespan of *Anopheles* vectors.

**Methods:**

A two-arm cluster-randomized controlled trial (cRCT) was conducted in Western Province Zambia. Seventy clusters of 250–350 households were assigned (1:1) by restricted randomization to an intervention arm (ATSB) or control arm (no ATSB) in the context of standard of care vector control (insecticide-treated nets and/or indoor residual spraying). Two ATSB stations (Westham Sarabi, 0.11% dinotefuran w/w) were maintained on exterior walls of eligible household structures for a 7-month deployment period (December-June) during the high malaria transmission season. The primary outcome was clinical malaria incidence among two consecutive seasonal cohorts of children aged 1–14 years, followed-up monthly from January-June in 2022 and 2023. Secondary outcome was *Plasmodium falciparum* prevalence among individuals aged over six months. Analysis compared clinical malaria incidence and prevalence between arms among the intention-to-treat population.

**Results:**

ATSB coverage, assessed by cross-sectional survey, was 98.3% in March–April 2022 and 89.5% in March–April 2023. 4494 children contributed any follow-up time to the cohort, with 2313 incident malaria cases in the intervention arm (1.28 per child per six-month transmission season), and 2449 in the control arm (1.38 per child-season). The incidence rate ratio between the two arms was 0.91 (95% CI 0.72–1.15, *p* = 0.42). 2536 individuals participated in cross-sectional surveys, with prevalence of *P. falciparum* 50.7% in the intervention arm and 53.5% in the control arm. The odds ratio between the two arms was 0.89 (95% CI 0.66–1.18, *p* = 0.42). Secondary covariable-adjusted and subgroup analyses did not substantially alter the findings. No serious adverse events associated with the intervention were reported.

**Conclusions:**

Two ATSB stations deployed per eligible structure for two consecutive transmission seasons did not result in a statistically significant reduction in clinical malaria incidence among children aged 1–14 years or in *P. falciparum* prevalence in rural western Zambia. Further studies are needed to assess the efficacy of ATSB stations in different settings and with different deployment strategies.

**Trial registration:**

The trial is registered with Clinicaltrials.gov (NCT04800055).

**Supplementary Information:**

The online version contains supplementary material available at 10.1186/s12936-024-05175-8.

## Background

Progress made against malaria since the early 2000s is under threat from various factors such as insecticide resistance and outdoor biting *Anopheles* spp. populations, necessitating new tools and approaches for vector control [1]. Attractive targeted sugar bait (ATSB) stations are one of several new vector control tools under development and evaluation, which could potentially mitigate the threats from residual malaria transmission. ATSB leverage mosquitoes’ natural sugar-feeding behaviour through an “attract and kill” approach, by providing foraging mosquitoes with a sugar source which includes an ingestion toxicant.

Early ATSB concepts delivered an oral insecticide through spraying of vegetation with a solution made from locally available fruits and boric acid [2, 3], or delivery of sugar bait solution inside households by improvised devices [4, 5]. Spraying vegetation with ATSB solution in Mali led to reduction in *Anopheles gambiae *sensu lato (*s.l*.) abundance by 90% [2], and a similar approach in Israel reduced density of female *Anopheles sergentii* by over 95% [3]. These promising studies led to development of the Sarabi ATSB device by Westham Ltd. (Hod-Hasharon, Israel) which includes date syrup as an attractant, dinotefuran, and Bitrex (to deter human ingestion) behind a perforated membrane which allows mosquito feeding but prevents access by non-target organisms. The manufacturer has determined that the Sarabi ATSB retains attractancy for six months (Amir Galili, personal communication).

Proof of concept studies with prototype Sarabi stations containing attractive sugar bait without toxicant (attractive sugar bait, ASB) demonstrated that local *Anopheles* species in Mali and Zambia will feed on ASB stations in the natural environment [6, 7]. In Mali, a trial among 14 villages found use of a Sarabi ATSB station significantly reduced female *An. gambiae s.l.* abundance, the proportion of female *An. gambiae s.l.* which had experienced three or more egg-laying cycles, and the entomological inoculation rate (EIR) [6].

Data from the Mali ATSB plant-spraying study and Sarabi ATSB station trial were utilized in mathematical models to predict impacts on malaria transmission. These models indicated that the feeding rates on ATSB and entomological impacts seen in field studies of ATSB were expected to translate into large reductions in malaria transmission even in the context of existing high coverage of ITNs [8, 9].

With a view to generating evidence of ATSB efficacy that could support establishing a new malaria vector control class with World Health Organization (WHO) recommendation, as well as contribute to the prequalification of the Sarabi ATSB product, a master protocol was developed for three similar but stand-alone Phase III trials of ATSB in Mali, Kenya and Zambia [10]. The three trial settings vary in their primary vector, seasonality, and housing density, but are similar in design, intervention deployment, and primary outcome assessment [10]. This study presents results from the first cluster-randomized controlled trial of ATSB efficacy against malaria burden. The trial was conducted over two years in western Zambia, with the primary endpoint the effect of ATSB stations on clinical malaria incidence in children aged 1–14 years during two seasons of deployment, and a secondary outcome the effect on malaria parasite prevalence in the peak transmission season among individuals aged over six months.

## Methods

### Study design

A two-arm cluster-randomized controlled trial was conducted in Kaoma, Luampa, and Nkeyema districts of Western Province, Zambia. This region experiences seasonal malaria transmission from January to June, with a peak in April–May. The study area does not have seasonal malaria chemoprevention programs. The dominant malaria vector species is *Anopheles funestus*, which has been shown to bite humans both inside houses and outdoors in adjacent peri-domestic spaces [7]. Clusters are sparsely populated, with median 0.36 domestic structures per hectare. Nectar-producing plants at the site include mango and evergreen trees, cassava, sweet potato, blackjack and coffeeweed. Further description of the study site is available elsewhere [11]. The full trial protocol has been published [10].

**Fig. 1:**
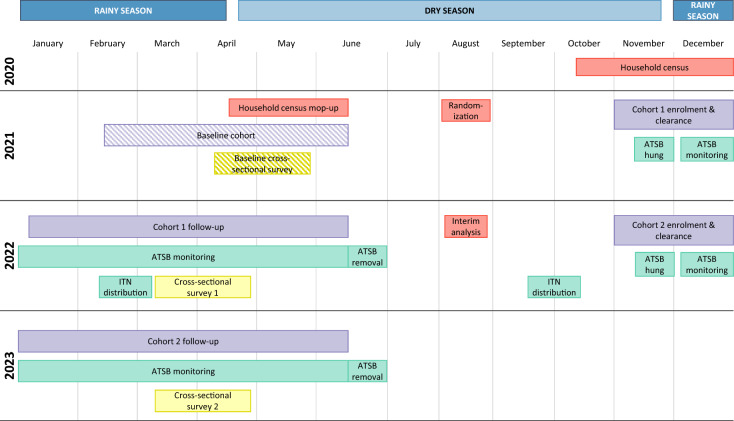
Trial timeline

A household census was conducted in October-December 2020, with a mop-up census in April-June 2021 targeted to incomplete or missed areas (Fig. 1). A K-means algorithm was used to draft initial boundaries for 85 clusters of 250–350 households, which were further refined using satellite imagery. Following baseline assessments, including parasite prevalence in 85 clusters, 70 clusters were retained for the trial. Clusters dropped were characterized by poor accessibility, high refusal, or low baseline malaria prevalence. The trial used a ‘fried egg’ design to prevent contamination, whereby outcome assessment was limited to households within the cluster ‘core’. Both the cluster core and households in a 600-m buffer area surrounding the core were assigned to receive intervention or control. Epidemiological outcomes were assessed through a cohort study and household surveys.

### Participants

Participants were recruited into two seasonal cohorts from 22 November—7 December 2021, and 21 November—5 December 2022. Census lists were used to select 35 households in each cluster each year by simple random sampling of eligible households without replacement; eligible households were those with a resident child aged from 12 months to 14 years. One child was randomly selected in each selected household. Eligible cohort participants were children aged 1–14 years at enrolment living in the cluster core, and testing negative for malaria by histidine rich protein-2 rapid diagnostic test (RDT: SD Bioline Malaria Ag P.f, Standard Diagnostics, South Korea, and Abbott Bioline Malaria Ag P.f, Abbott, USA) two weeks following receipt of a full dose of artemether lumefantrine (AL) at enrolment (see Procedures, below) [10]. Exclusion criteria for the cohort were pregnancy (assessed by question at enrolment and each follow-up visit), history of contraindication to AL, severe illness, consent or assent refusal, or plans to relocate from their household in the next six months. Children permanently relocating outside their enrolment cluster were excluded from subsequent follow-up visits. Children absent on one or more follow-up visits had follow-up time censored to one month prior to the next successful visit [12].

Household survey participants were selected in each cluster each year by simple random sample from the census list in April–May 2022 and 2023. One individual in each selected household was randomly selected from the roster of usual household residents to test for a *Plasmodium falciparum* infection by RDT; children aged under six months of age or enrolled in the cohort study were ineligible. There was no upper age limit for participation in the household survey. The target recruitment was 20 households per cluster, per survey year.

Written informed consent was obtained from household heads in intervention clusters for installation of ATSB stations on eligible structures. Written informed consent was obtained from parents or guardians of cohort participants, together with written assent from cohort participants aged 7–14 years. Written informed consent was received from household heads for cross-sectional survey participation, and from the household resident (or their parent/guardian) randomly selected for RDT in the survey, together with written assent from selected children aged 7–17 years.

### Randomization

Restricted randomization was conducted by an independent statistician to assign clusters in a 1:1 ratio to distinct arms. Restricted randomization criteria were baseline malaria prevalence, ITN use, IRS receipt, number of households in cluster, health facility presence within cluster, and use of sites within the cluster for baseline entomological surveillance activities (Table S1). An initial 500,000 random allocation sequences were generated, of which 148 met all the restriction criteria. One randomization sequence was selected at random. Assignment of arms A and B to intervention or control was determined by coin toss. Allocation was masked from the analyst during data cleaning and primary outcome analysis.

### ATSB intervention

**Fig. 2:**
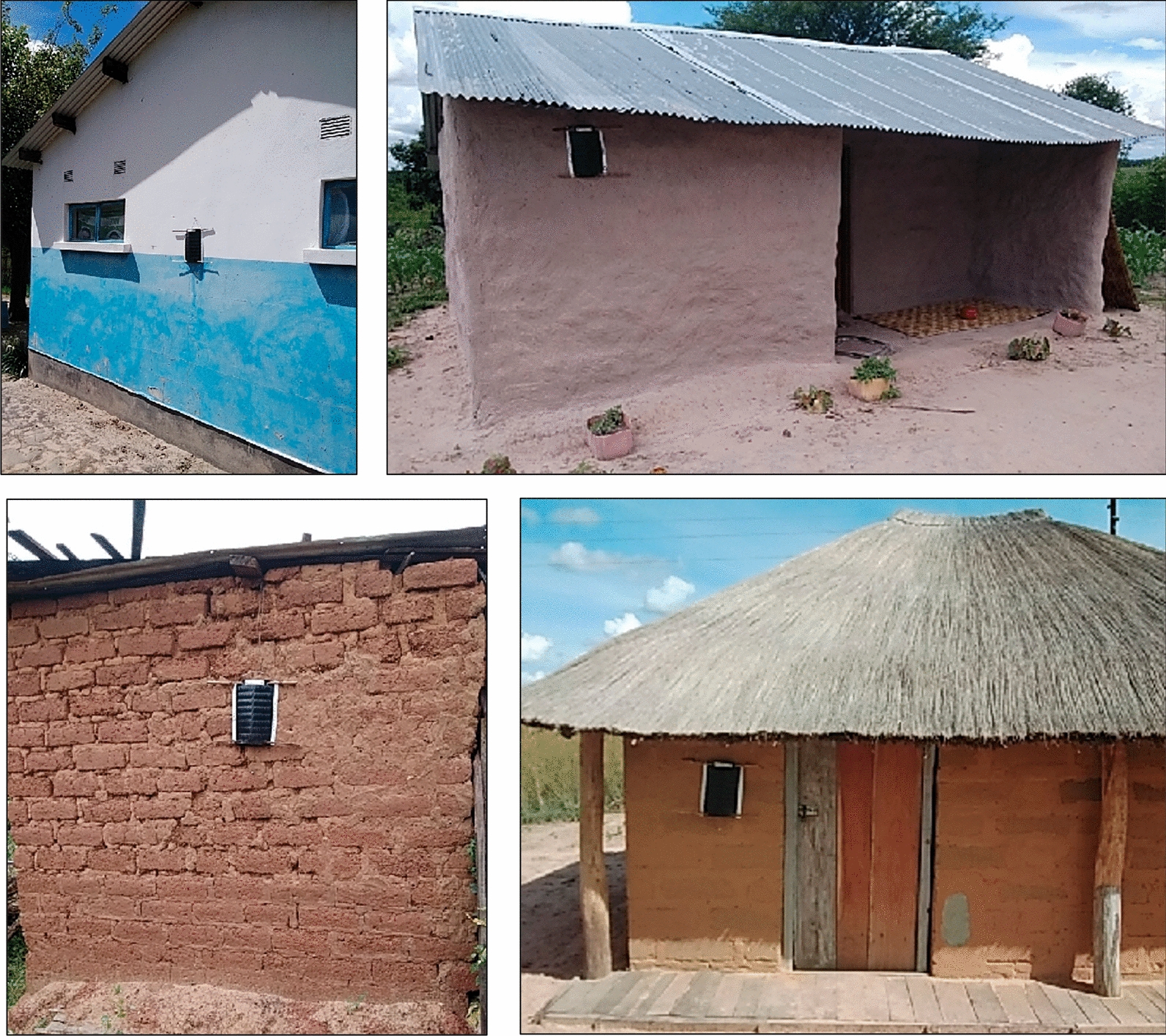
Examples of ATSB installed on structure walls

In intervention clusters, two ATSB stations (Sarabi version 1.2) were installed on each eligible structure of a consenting household (Fig. 2). Eligible structures were sleeping structures or kitchens also used for sleeping, with minimum of three complete walls at least 1 m high, and a complete roof. Toilets, bathing shelters, drying racks, tobacco drying sheds, structures with visible insect infestation, under construction or collapsed were all ineligible. ATSB stations were placed on opposite exterior walls of the structure unless adjacent walls provided better protection, at 1.8 m above ground level and under eaves where possible to protect from rain and sun (Fig. 2). ATSB station placement was informed by pilot studies in Mali [13]. Installation campaigns were conducted from 1–13 November 2021 and 31 October to 12 November 2022. Trained community members consented households and installed ATSB stations, recorded structure location and station barcodes using a bespoke digital data collection tool (Commcare, Dimagi, Cambridge MA) on android mobile devices. Community sensitization activities were conducted in intervention and control clusters prior to installation campaigns.

Trained community members visited installed ATSB stations at least once every two months during the trial season (December-June) to ensure ongoing high intervention coverage. This encompassed installing ATSB stations on newly built or newly eligible structures, replacing damaged or missing stations, and removing stations from collapsed structures or those which were no longer eligible. Full details of the ATSB station deployment, monitoring, removal, and disposal procedures are described elsewhere [14].

### Standard of care vector control

The standard of care for malaria vector control in areas with ongoing malaria transmission was universal coverage with ITN and/or IRS. During the trial period, Zambia National Malaria Elimination Centre (NMEC) used a mosaic approach to assign health facility catchments to receive either IRS or ITN during annual microplanning exercises that determined the most appropriate intervention for each catchment area [15]. As such, the trial site included both IRS- and ITN-targeted areas, but because study cluster boundaries were drawn without direct consideration of health facility catchments, most study clusters contained a mixture of households that received IRS and households that received ITNs. In year one, IRS was conducted by the NMEC in parts of 42 clusters (24 intervention, 18 control) from 15 October 2021 to 4 February 2022 using clothianidin-deltamethrin (Fludora^®^ Fusion, Bayer). In year two, IRS was conducted in parts of 21 clusters (14 intervention, 7 control) from 18 October to 31 November 2022 using clothianidin-deltamethrin. The vector population at the study site is susceptible to clothianidin, but has high pyrethroid resistance [16].

ITN distributions in the study area were conducted from 25 February to 17 March 2022, and 19 September to 14 October 2022. The first distribution provided one ITN (deltamethrin-impregnated, PermaNet^®^ 2.0, Vestergaard) to each household across all 70 clusters (28,908 nets) irrespective of IRS targeting, due to high community demand for nets. The second ITN distribution (59,051 nets) targeted the 49 clusters which were not selected or partially selected to receive IRS by NMEC 2022 microplanning, allocating one ITN (pyrethroid piperonyl butoxide [PBO] impregnated, Veeralin^®^LN, VKA Polymers) for every two residents in the household.

### Procedures

At enrolment into the cohort, children were provided with a full dose of AL to clear any preexisting *P. falciparum* infection. Participants were visited 14 days after enrolment to confirm parasite clearance; all children were tested by RDT, then duplicate thick blood films for microscopy if the RDT was positive. Those who missed the clearance visit or were positive by microscopy had a final chance to demonstrate clearance by being RDT negative at the first monthly follow-up visit 28 days later. Children who confirmed clearance proceeded into the cohort, where six scheduled visits were made to the child’s home at 28 day intervals during the transmission season (January–June). At each visit, children were assessed for fever (axillary temperature ≥ 37.5 °C or report of fever in previous 48 h) and those with fever tested by RDT. Those with a positive RDT result received a full course of AL according to national guidelines. A questionnaire was completed at each visit to collect information on ITN ownership and use, IRS within the previous 12 months, child’s sleeping structure construction, and child’s health in the previous month. Demographic information was collected at enrolment.

Cross-sectional household surveys took place March–April 2022 and 2023, concurrent with the peak transmission season. One household member was randomly selected to be tested for *P. falciparum* infection by RDT; those with positive RDT result were provided with a full course of AL in accordance with national guidelines. Information on ITN ownership and use, IRS within the previous 12 months, and inspection of household structures for ATSB stations to assess their presence and condition were completed during the visit, together with collection of other household-level indicators using a standardized questionnaire administered to the head of household by a trained interviewer.

### Outcomes

The primary outcome for the study was incidence of symptomatic malaria in children aged 12 months to 14 years, defined as either reported fever in the previous 48 h or axillary temperature ≥ 37.5 °C, and a positive RDT result. The secondary epidemiological outcome was prevalence of *P. falciparum* infection as detected by RDT among cross-sectional household survey participants aged at least six months. Entomological outcomes [16] and cost effectiveness are reported elsewhere (Mancuso et al., in preparation).

Adverse events (AEs) and severe adverse events (SAE) were monitored through passive and active data collection. Adverse events related to ATSB exposure and AL receipt were collected at cohort follow-up visits, with additional information on ATSB exposure AEs collected through the household survey. SAEs were defined as any death of a cohort participant or any ingestion of bait from ATSB stations in the general community.

### Statistical analysis

A full statistical analysis plan for the trial is published elsewhere [12]. Sample size calculations followed standard cluster randomized trial methods [17]. Briefly, the trial was designed to detect a 30% reduction in cumulative malaria case incidence over two seasonal cohorts (six months of follow-up each study year), with sample size of 35 clusters per arm, 80% power and alpha 0.05. We assumed a baseline incidence of 0.50 events per six-month transmission season and coefficient of variation of 0.4. The cohort had target recruitment of 35 children per cluster per season and assumed 34% loss to follow up (2450 children enrolled each year). The cross-sectional survey was designed to detect a 30% reduction in prevalence with 90% power, two-tailed alpha of 0.05, intracluster correlation coefficient of 0.10 and baseline prevalence of 50%. The survey had target recruitment of 20 individuals per cluster per year and assumed 20% non-response (1400 individuals selected each year).

Statistical analyses were done in R version 4.2.2 (R Foundation for Statistical Computing, Vienna, Austria). To generate incidence by arm, incidence was generated for each cluster in the relevant arm, then mean of cluster-level incidence estimated together with 95% confidence intervals. The primary unadjusted intention-to-treat (ITT) analysis was a comparison of clinical malaria incidence between the two arms, using a generalized linear model (GLM) framework with Poisson likelihood and log link function, including random intercepts for each study cluster. The secondary outcome, infection prevalence, was analysed among the ITT population using an un-adjusted GLM with a Bernoulli likelihood and logit link function, including random intercepts for each cluster.

Secondary, covariable-adjusted analyses were completed for both the primary and secondary outcome, using the following covariables: baseline infection prevalence, implementation year, age, rainfall anomaly, ITN use, receipt of IRS at household, and a single analysis including all covariables included in restricted randomization. Subgroup analyses were conducted for the following covariables: gender, age, baseline prevalence, housing type (closed vs. non-closed eaves), and rainfall (one month lagged total rainfall, classified as high [≥ site mean] or low). Assessment of the homogeneity of treatment effect by subgroup was conducted by inclusion of the treatment, subgroup variable and their interaction term as predictors in primary and secondary outcome models.

Per-protocol analyses were done for the primary and secondary outcome, defining the per-protocol population at the cluster-level from the specific year’s household survey data according to two adherence standards; (i) coverage of ATSB stations (intervention clusters where ≥ 80% of eligible structures had ≥ 2 ATSB stations in any condition and control clusters with 0% coverage on eligible structures), and (ii) coverage of ATSB stations in good condition (intervention clusters with ≥ 50% of eligible structures having ≥ 2 ATSB stations that do not meet pre-defined replacement criteria [holes, mold, leaks, depletion, dirt] [14] and control clusters with 0% coverage on eligible structures).

Post-hoc analyses examined time to first clinical malaria case between arms using a Cox proportional hazards model with cluster shared frailty [18, 19], and sub-group analysis among low structure density clusters (those where total structures enumerated in the cluster core divided by cluster core size in hectares was less than 1 structure/hectare) and larger clusters (≥ 1 structures/hectare) (Fig. S1).

The trial primary outcome was assessed with consideration for multiple hypothesis testing due to pre-planned interim analysis [12]. All other p-values reported are nominal and do not reflect any adjustments for multiple hypothesis testing. An independent Data Safety and Monitoring Board was established for the Zambia trial site. The trial is registered with ClinicalTrials.gov, NCT04800055.

## Results

**Fig. 3:**
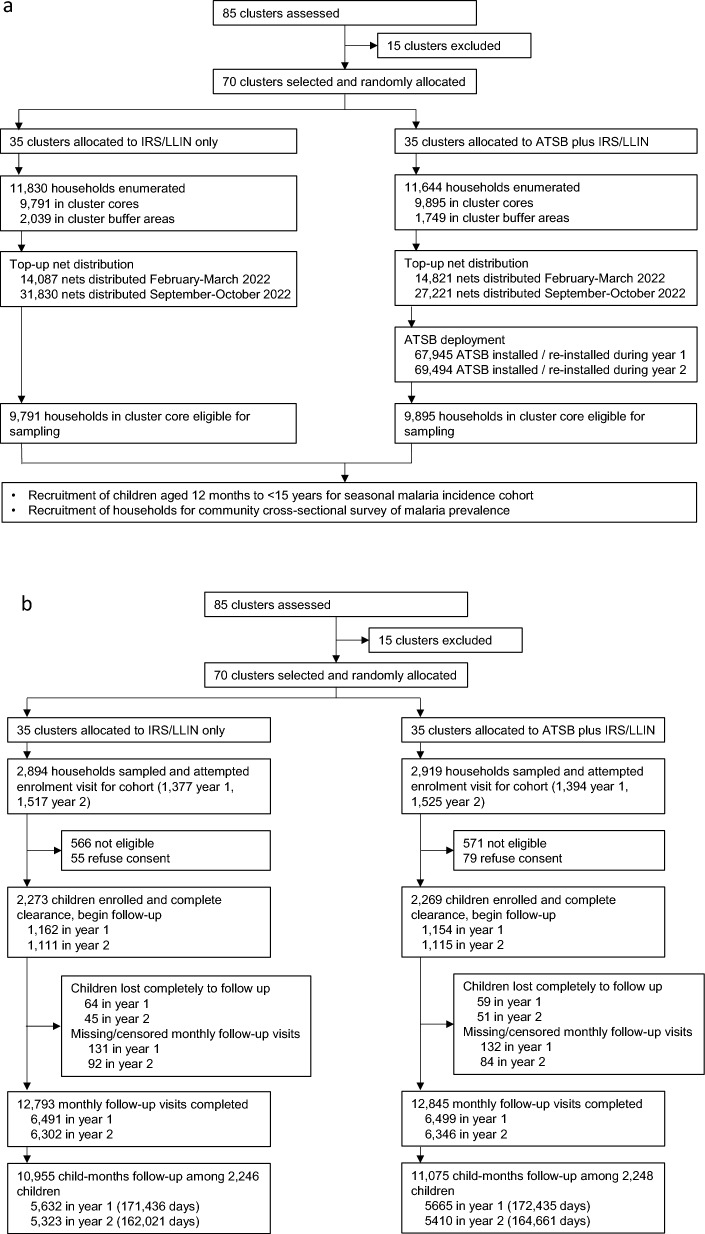

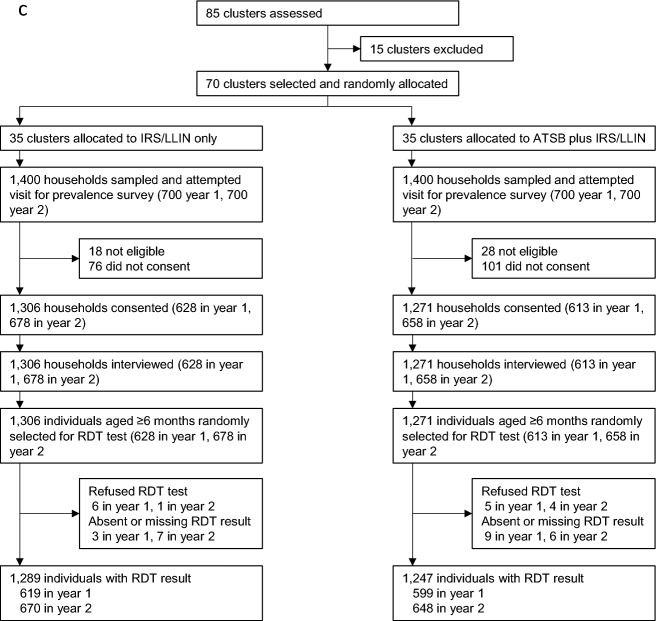
Trial profile describing (**a**) cluster assignment and intervention deployment; (**b**) recruitment of children for seasonal cohort; and (**c**) participation in cross-sectional survey

A total of 23,474 households were enumerated within the study clusters, with 19,686 households located in the cluster cores (Fig. 3). In year 1 of the trial 41,695 ATSB stations were deployed during the initial installation campaign (1–13 November 2021), with an additional 26,250 ATSB stations deployed on newly built structures or to replace damaged ATSBs in the period until hang-down began on 15 June 2022. In year 2, 41,982 ATSB stations were initially deployed (31 October to 12 November 2022), with 27,512 additional ATSB stations deployed as replacements or on newly built structures in the period until hang-down was started on 15 June 2023.

The first seasonal cohort enrolled 2316 participants in November 2021, 2293 of whom contributed any follow-up time until the cohort end in June 2022. The second seasonal cohort enrolled 2226 children in November 2022, and 2201 participants contributed any follow-up time up to the cohort end in June 2023. Total follow-up time at risk accrued over both seasonal cohorts was 22030 child-months. Loss to follow-up was similar between arms Fig. 3. Characteristics of cohort participants are described in Table 1.

**Table 1:** Study population characteristics

	Overall	Control arm	Intervention arm
Cluster description			
Number of clusters	70	35	35
Total population in core and buffer cluster areas at trial commencement	122023	60518	61505
Mean population in core area of cluster (range)	1461 (894–2397)	1454 (894–1903)	1467 (920–2397)
Mean size of cluster core in hectares (range)	1435 (66–7570)	1389 (86–7570)	1481 (66–5057)
Baseline cross-sectional survey (March–April 2021) (limited to 70 clusters continuing to trial)			
Mean age, years (95% CI)	27.7 (26.3–28.8)	27.1 (25.4–28.8)	28.2 (26.5–29.9)
Malaria infection prevalence (95% CI)	51.9% (48.7–55.0)	52.3% (47.7–56.9)	51.5% (46.9–56.1)
Children (age 1–14 years) at cohort enrolment (November 2021 & 2022)			
Proportion of children < 5 years	33.1%; 1489/4494	33.2%; 746/2246	33.1%; 743/2248
Proportion of female children	51.3%; 2304/4494	51.2%; 1150/2246	51.3%; 1154/2248
Net use the night before enrolment	55.9%; 2509/4491	57.3%; 1285/2244	54.5%; 1224/2247
Child’s household received IRS in previous 12 months	30.5%; 1364/4470	28.0%; 626/2236	33.0%; 738/2234
Net use the previous night or household received IRS	71.5%; 3214/4494	71.3%; 1602/2246	71.7%; 1612/2248
Cross-sectional household survey (March–April 2022 & 2023)			
ITN ownership (at least 1 ITN in the household)	95.2%; 2415/2536	96.5%; 1244/1289	93.9%; 1171/1247
IRS received in 12 months prior to survey	29.7%; 749/2522	27.7%; 356/1286	31.·8%; 393/1236
Own at least 1 ITN or household received IRS	96.7%; 2450/2534	97.3%; 1254/1289	96.·1%; 1196/1245
Proportion of respondents used ITN last night	72.9%; 1849/2535	75.0%; 967/1289	70.·8%; 882/1246
Proportion of eligible structures at surveyed households with ≥ 2 ATSB	N/A	0%; 0/2429	93.·1%; 2217/2381

During the total 12 months follow-up over two malaria transmission seasons, we detected 4762 clinical malaria cases during the scheduled monthly follow-up visits. The mean clinical malaria incidence was 1.28 cases per child per six-month transmission season (95% CI 1.06–1.50) in the intervention arm, and 1.38 cases per child per 6 month transmission season (95% CI 1.18–1.57) in the control arm (unadjusted IRR 0.91; 95% CI 0.72–1.15; p = 0.42; Table 2). Observed coefficient of variation in the control arm was 0.35.

**Table 2:** Clinical malaria incidence in children aged 1–14 years per 6-month transmission season of follow-up

	Control arm			Intervention arm			Unadjusted incidence rate ratio (95% CI)	p value
	Number of clinical malaria episodes	Child-months of follow-up	Incidence per child per 6 month season (95% CI)	Number of clinical malaria episodes	Child-months of follow-up	Incidence per child per 6 month season (95% CI)		
Overall	2449	10955	1.38 (1.18–1.57)	2313	11075	1.28 (1.06–1.50)	0.91 (0.72–1.15)	0.42
Year 1	1145	5632	1.25 (1.07–1.44)	1080	5665	1.17 (0.95–1.40)	0.89 (0.68–1.18)	0.43
Year 2	1304	5323	1.50 (1.30–69)	1233	5410	1.39 (1.18–1.60)	0.91 (0.73–1.13)	0.39

Clinical malaria incidence was slightly higher in both arms in the second year, but there was no significant difference between arms by year. Interactions between covariables (baseline prevalence, age, gender, housing type) and arm were not significant. There was evidence for an interaction between lagged rainfall anomaly and arm (p = 0.002); sub-group analysis indicates a slightly larger but still non-significant ATSB effect in areas with higher rainfall (IRR 0.77, 95% CI 0.57–1.04, p = 0.093, Table S2). Covariable-adjusted analyses (baseline prevalence, trial year, age, ITN use, IRS, and all randomization covariables) did not result in any change in ATSB effect size (Table S3). Unadjusted analysis using per-protocol populations yielded similar effect estimates to the primary unadjusted model output (Table S4).

In sub-group analyses by cluster structure density, there was a larger ATSB effect in the high structure density clusters (IRR 0.79, 95% CI 0.50–1.26, p = 0.33) than in the lower structure density clusters (1.05, 95% CI 0.85–1.30, p = 0.64), but effect size was not statistically significant in either sub-group Table 3. Median time to first clinical case was longer in the ATSB arm than control arm (141 days vs 119 days) but did not reach statistical significance (Hazard Ratio 0.88, 95% CI 0.66–1.16, p = 0.36).

**Table 3:** Post-hoc subgroup analysis of ATSB effect on clinical malaria incidence among children aged 1–14 years in clusters with low structure density (those with density of less than of 1 structure/hectare) and clusters with high structure density (≥ 1 structures/hectare)

Population	Control arm			Intervention arm			Unadjusted incidence rate ratio (95% CI)	p value
	Number of clinical malaria episodes	Child-months of follow-up	Incidence per child per 6 month season (95% CI)	Number of clinical malaria episodes	Child-months of follow-up	Incidence per child per 6 month season (95% CI)		
Intention-to-treat	2449	10955	1.38 (1.18–1.57)	2313	11075	1.28 (1.06–1.50)	0.91 (0.72–1.15)	0.42
Subgroup: high structure density clusters	617	3266	1.16 (0.90–1.43)	669	4601	0.90 (0.69–1.11	0.79 (0.50–1.26)	0.33
Subgroup: low structure density clusters	1832	7780	1.44 (1.23–1.66)	1644	6566	1.54 (1.28–1.80)	1.05 (0.85–1.30)	0.64

Overall prevalence of *P. falciparum* infection by RDT in the household survey was 50.7% in the ATSB arm and 53.5% in the control arm but did not differ significantly by arm (OR 0.89, 95% CI 0.66–1.18, p = 0.42). Prevalence was lower in the ATSB arm in both trial years, but there was no significant difference by year between arms (Table 4). Interactions between covariables (baseline prevalence, age, gender, housing type, rainfall anomaly) and arm were not significant. Covariable-adjusted analyses did not result in meaningful change in estimates of the effect size for ATSB (Table S5). Analyses using the per-protocol population also did not result in meaningful changes to the estimates of effect compared to the primary unadjusted ITT model (Table S6).

**Table 4:** Malaria prevalence among participants aged > 6 months in cross-sectional household surveys conducted at peak transmission season

	Control arm		Intervention arm		Unadjusted odds ratio	95% CI	p value
	N	Prevalence	N	Prevalence			
Overall	1289	53.5%	1247	50.7%	0.89	0.66–1.18	0.42
Year 1	619	55.3%	599	50.7%	0.84	0.58–1.21	0.34
Year 2	670	51.8%	648	50.6%	0.95	0.71–1.27	0.73

Four malaria deaths occurred among cohort participants during the trial implementation: one in the ATSB arm and three in the control arm. No instances of human ingestion of bait were reported. All SAEs were determined by the independent DSMB as not trial related. Adverse event results were pooled from each cohort follow-up visit across both study years (Table S7). Compared to the control arm, the ATSB arm had higher reports of eye irritation (2.7% of child follow-up visits vs. 2.3%, p = 0.024), itching (5.5% vs. 4.8%, p = 0.016), and rash (8.1% vs. 6.9%, p < 0.001), but the DSMB determined that these AEs were not related to the intervention.

## Discussion

ATSB stations deployed in western Zambia at the rate of two per eligible structure resulted in a non-significant 9% reduction in clinical malaria incidence over two transmission seasons, and a non-significant 5.2% relative reduction in malaria prevalence. The ATSB stations deployed at the study site according to protocol did not meet the targeted 30% reduction in malaria burden to be considered a public health benefit by WHO's Vector Control Advisory Group [20]. Sub-group analysis indicates a 21% reduction in clinical incidence among clusters with high structure density, suggesting differences in effect size across the trial site.

The trial was characterized by a high programmatic coverage of the intervention throughout two transmission seasons, with a community-based programme of bait station monitoring to identify and replace damaged bait stations according to predetermined criteria for replacement. While bait stations were retained at as high coverage as feasible during the trial, on average 14% of bait stations were estimated to meet criteria for replacement due to damage at any time [14]. It is known that bait stations in good condition (not meeting replacement criteria) remain bio-efficacious in laboratory testing against *Anopheles* after a seven-month deployment on structures in the trial area in Zambia [21], but there is a lack of evidence on the ability of damaged bait stations to kill mosquitoes, or the extent or type of damage that reduces bait station efficacy.

The study population in western Zambia live in widely dispersed settlement patterns [11]. The ATSB intervention is hypothesized to provide community-level rather than individual-level protection, and it is plausible that bait station density in space is a determinant of efficacy.

Presently, there is no target spatial density for deployment associated with the Sarabi bait station, nor with ATSB deployment more broadly. In agricultural uses, attract-and-kill interventions often define the spatial density (e.g., lures per hectare) required for effect on the target insect [22]. Secondary analysis of trial data is ongoing to further explore the relationship between bait station spatial density and efficacy (Mancuso et al*.*, in preparation). Sub-group analysis suggests a larger, though non-significant, effect of ATSB on clinical malaria incidence in clusters with relatively higher structure density. There may be other unmeasured systematic differences between clusters classified as high or low structure density, including but not limited to levels of natural sugar availability, which may be associated with vector feeding rates on the ATSB stations, size of settlements within clusters, and proximity of vector breeding sites.

While intention-to-treat analysis of the primary outcome considered multiple hypothesis testing due to a planned interim analysis, secondary and sub-group analyses did not. As such, the nominal p-values may be a serious underestimate of the true type-I error probability for specific hypothesis tests. Consequently, secondary and sub-group analyses should be treated with caution and considered as hypothesis-generating findings, not definitive confirmations of efficacy.

Results from a pre-trial ASB feeding study [7] suggested that the study site was characterized by rates of vector feeding on ASB (sham ATSB stations) estimated to be sufficient to achieve the target reduction in clinical malaria incidence [9]. Trial results do not align with these, or other, modelled outcomes from several studies estimating the impact of bait stations on entomological and epidemiological outcomes [8, 9]. In those models, which were parameterized using results of entomological field trials in the Sahelian region of Mali, the rate at which mosquitoes fed on bait stations was the key determinant of excess mosquito mortality, which drove estimates of ATSB impact. While this relationship between vector bait station feeding rates and mosquito mortality is likely to be true in any transmission setting, it is possible that the specific rates required to achieve desired reductions in malaria transmission could vary substantially in different contexts, and higher rates of bait station feeding may be required to achieve similar public health outcomes in western Zambia. Furthermore, there are relatively limited data available describing bait station feeding rates, which are typically estimated by sampling mosquitoes from CDC light traps set in close proximity to the structures where bait stations are installed. Sampling vectors from locations where bait stations are easily accessible may lead to an overestimation of general feeding rates in the entire vector populations relevant to local malaria transmission. This overestimation bias may be exacerbated in settings like western Zambia, where the dominant vector species *Anopheles funestus* has poorly characterized larval habitats that are nonetheless likely to be numerous and relatively widespread [23–25] in relation to human settlements that are well dispersed and have low structure density, as well as unknown natural sugar feeding behaviours. Accordingly, the validity of this light trap sampling approach to accurately estimate general bait station feeding rates in the broader vector population should be investigated further.

Recent evidence from Kenya has highlighted *An. funestus* biting in the morning after people have exited their nets for the day [26] and biting in schools until 11:00am [27], while data from Tanzania report a longer average lifespan of *An. funestus* compared to *Anopheles arabiensis* [28]. These characteristics of *An. funestus* may be contributing to the higher-than-expected malaria transmission in western Zambia, where *An. funestus* is the clear primary vector [7]. Further data describing *An. funestus* blood- and sugar-feeding behaviour, including preferred sources of natural sugar and the extent of sugar feeding near adult emergence/breeding sites versus potential blood-feeding sites will help to further understand the potential of ATSB in settings such as Zambia with high availability of competing natural sugar sources and high vectorial capacity.

This trial was designed to assess the impact of ATSB stations against a background of high coverage routine vector control. In Zambia this entailed a mosaic of IRS and ITNs. IRS during the trial period used a combination product with deltamethrin (a pyrethroid) and clothianidin (a neonicotinoid); however, IRS campaigns using this product have reported suboptimal malaria control in Uganda [29] and in Zambia [30]. Due to global COVID-19 related shipping delays, our planned distribution of ITNs was delayed. An interim distribution of pyrethroid ITNs was conducted partway through the first year of the trial. The full distribution of PBO ITNs took place between years one and two, further boosting ITN use in the study population. In this context, there was no evidence for ATSB effect modification by IRS status or use of ITN (of either type) among the cohort or household survey populations, with routine vector control being balanced at high population coverage between arms.

The cohort study component of this trial had low loss-to-follow-up (5% enrolled children were lost to follow-up), and both cohort and household survey components exceeded the required sample size. Malaria transmission in the study site in western Zambia was higher than expected, especially given the high level of malaria control efforts in the area. Clinical malaria incidence in the cohort study was substantially higher in the trial (average 2.75 clinical cases per child per year in the control arm) than estimated in the sample size from passive surveillance data (estimated from routine data to be 0.50 clinical cases per child per year). Consequently, our trial was estimated to have 97% power to detect a 30% reduction in incidence between arms with the observed incidence of 2.75 clinical cases per child per year in the control arm and observed coefficient of variation of 0.35. Other studies comparing incidence estimates from passive surveillance and active cohort have found double or three-times higher incidence in the cohort being actively monitored [31, 32].

Trials of Westham Sarabi ATSB stations in Mali and Kenya concluded in January 2024 and March 2024 respectively, and results are in preparation. The Mali and Kenya trial settings differ from the Zambia setting in their malaria transmission seasonality, vector bionomics, human settlement patterns and density, and natural sugar availability. ATSB efficacy estimates from these trial sites along with planned meta-analysis of all three sites, will further elucidate the potential future public health benefit of ATSB interventions, and may indicate potential drivers of efficacy, suitable settings for ATSB stations, or variations in deployment strategy that may improve impact.

## Supplementary Information

Supplementary Material 1

## Data Availability

De-identified data are available from the corresponding author on reasonable request. Following publication of forthcoming secondary analyses of trial data, the deidentified trial dataset will be posted on a public repository.
