# Supplementary material for: Efficacy of attractive targeted sugar bait stations against malaria in Western Province Zambia: epidemiological findings from a two-arm cluster randomized phase III trial
Source: Malar J. 2024 Nov 15;23:343. doi: 10.1186/s12936-024-05175-8 (PMC11566550; doi:10.1186/s12936-024-05175-8)
Supplement: Supplementary file 1 — Supplementary Material 1 [file 12936_2024_5175_MOESM1_ESM.pdf]

Supplementary information

Efficacy of Attractive Targeted Sugar Bait stations against malaria in Western Province, Zambia: epidemiological findings from a two-arm cluster randomized Phase III trial

Ruth A Ashton, Kochelani Saili, Chama Chishya, Handrinah Banda Yikona, Annie Arnzen, Erica Orange, Chanda Chitoshi, John Chulu, Titus Tobolo, Frank Ndalama, Irene Kyomuhangi, Willy Ngulube, Hawela Moonga, Jacob Chirwa, Laurence Slutsker, Joseph Wagman, Javan Chanda, John Miller, Kafula Silumbe, Busiku Hamainza, Thomas P. Eisele, Joshua Yukich, Megan Littrell

Table S1: Restricted randomization variables, p-value thresholds, and maximum mean difference/variance ration between arms that were permitted by defined threshold. 2

Figure S1: Map of trial clusters..3

Table S2 Sub-group analysis of ATSB effect on clinical malaria incidence among children aged 1-14 years...4

Table S3: Summary of covariate-adjusted ATSB effect estimates on clinical malaria incidence 4

Table S4: Summary of per-protocol analyses of ATSB effect estimates on clinical malaria incidence...4

Figure S2: Scatter plot of structures per hectare in the cluster core versus cluster core size in hectares. 5

Figure S3: Scatter plot among intervention clusters of ATSB station density versus structure density...6

Table S5: Summary of covariate-adjusted ATSB effect estimates on *P. falciparum* infection prevalence..7

Table S6: Summary of per-protocol analyses of ATSB effect estimates on *P. falciparum* infection prevalence..7

Table S7: Summary of adverse events by trial arm 7

Table S8: Summary of adverse events between children who received artemether-lumefantrine on the previous visit and those who did not receive treatment...8

Table S1: Restricted randomization variables, p-value thresholds, and maximum mean difference/variance ration between arms that were permitted by defined threshold.

Baseline characteristics	Statistical test	p-value threshold	Maximum Mean Difference / Variance Ratio
Cluster RDT prevalence	t-test	>0.75	1.02%
	F-test	>0.75	1.12
Health facility located within cluster (yes/no)	Chi-square test	>0.5	8.57%
Log (Number of households in cluster)	t-test	>0.5	0.04
	F-test	>0.5	1.26
Cluster-level proportion of population report sleeping under ITN on previous night	t-test	>0.5	4.21%
	F-test	>0.5	1.26
Cluster-level proportion of households report receiving IRS in previous 12 months	t-test	>0.5	4.48%
	F-test	>0.5	1.26

Restricted randomization was undertaken to minimize differences between arms on baseline characteristics. The following steps were completed as part of this process:

- 500,000 potential allocations of clusters to arm were generated
- Allocations with exactly 12 entomology clusters assigned to each arm were retained (99,061 potential allocations)
- Balance of baseline characteristics were checked according to statistical test thresholds listed in table S1. After applying all criteria in table S1 to the 99,061 potential allocations, 148 allocations remained
- Allocations remaining were checked for pairs of clusters either rarely or frequently allocated together. Two clusters were allocated together only 18% of the time. Among all other clusters, no pairs were allocated together more than 75% or fewer than 25% of the time. All 148 allocations remained eligible for final selection.
- One allocation among the 148 eligible was randomly selected
- Intervention and control arm were randomly assigned to the selected allocation

Figure S1: Map of trial clusters

Clusters used a 'fried egg' design whereby households within the cluster core and a 600m buffer area were assigned to receive the intervention or control. Outcome assessment was limited to households within the cluster core. Households within the buffer area were not eligible to participate in cohort or household survey. The area in the red square is enlarged for detail to illustrate the cluster core and buffer areas.

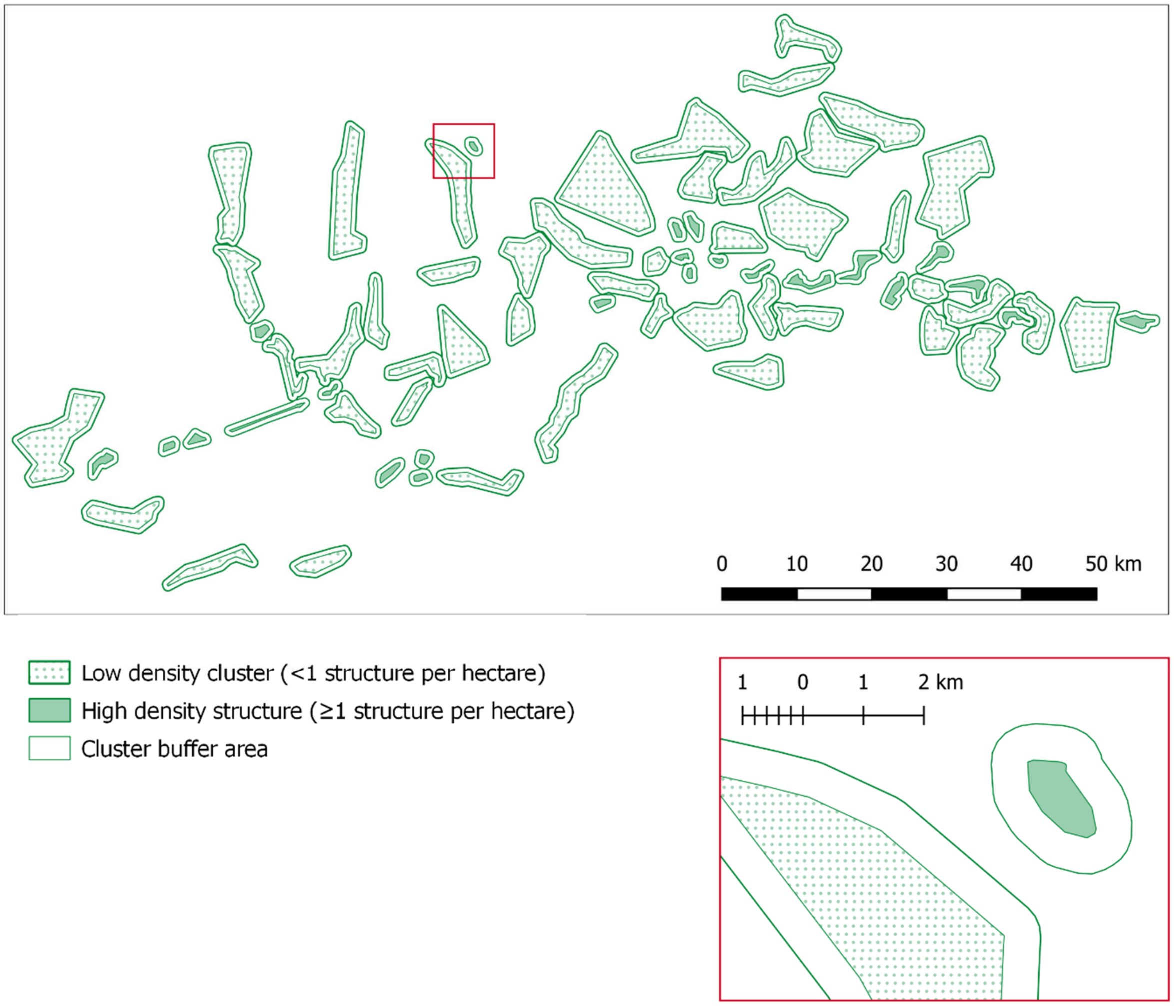

Table S2 Sub-group analysis of ATSB effect on clinical malaria incidence among children aged 1-14 years.

Sub-groups were defined as children resident in areas with lower than average rainfall (total prior month's rainfall anomaly at child's location over all visits <0), and children resident in areas with higher than average rainfall (total prior month's rainfall anomaly at child's location over all visits >0)

		ATSB effect		
**Model population**	**N**	**IRR**	**95% CI**	**p value**
Intention-to-treat	4944	0.91	0.72-1.15	0.422
Subgroup: low rainfall	2533	0.94	0.73-1.20	0.612
Subgroup: high rainfall	1961	0.77	0.57-1.04	0.093

Table S3: Summary of covariate-adjusted ATSB effect estimates on clinical malaria incidence

	**ATSB effect, covariate-adjusted model**		
**Covariable**	**Incidence rate ratio (IRR)**	**95% CI**	**p value**
Baseline cluster prevalence : low vs. high	0.94	0.76-1.17	0.600
Implementation year	0.91	0.72-1.14	0.481
Age: <5 years vs. 5-14 years	0.91	0.72-1.14	0.421
ITN use: never, sometimes, always	0.91	0.72-1.14	0.413
IRS at household	0.92	0.73-1.15	0.452
All restricted randomization covariates	0.92	0.75-1.13	0.439

Table S4: Summary of per-protocol analyses of ATSB effect estimates on clinical malaria incidence

		**ATSB effect, covariate-adjusted model**		
**Model population**	**N**	**IRR**	**95% CI**	**p value**
Intention-to-treat	4944	0.91	0.72-1.15	0.422
Per-protocol: intervention clusters with ≥80% of eligible structures had ≥2 ATSB in any condition	4363	0.89	0.71-1.13	0.345
Per-protocol: intervention clusters with ≥50% of eligible structures had ≥2 ATSB that did not meet replacement criteria	4180	0.91	0.72-1.15	0.419

Figure S2: Scatter plot of structures per hectare in the cluster core versus cluster core size in hectares.
Cluster structure density is calculated by counting total structures enumerated in the pre-trial census in the cluster core and dividing by cluster core area in hectares. Horizontal dashed line indicates structure density of 1 per hectare

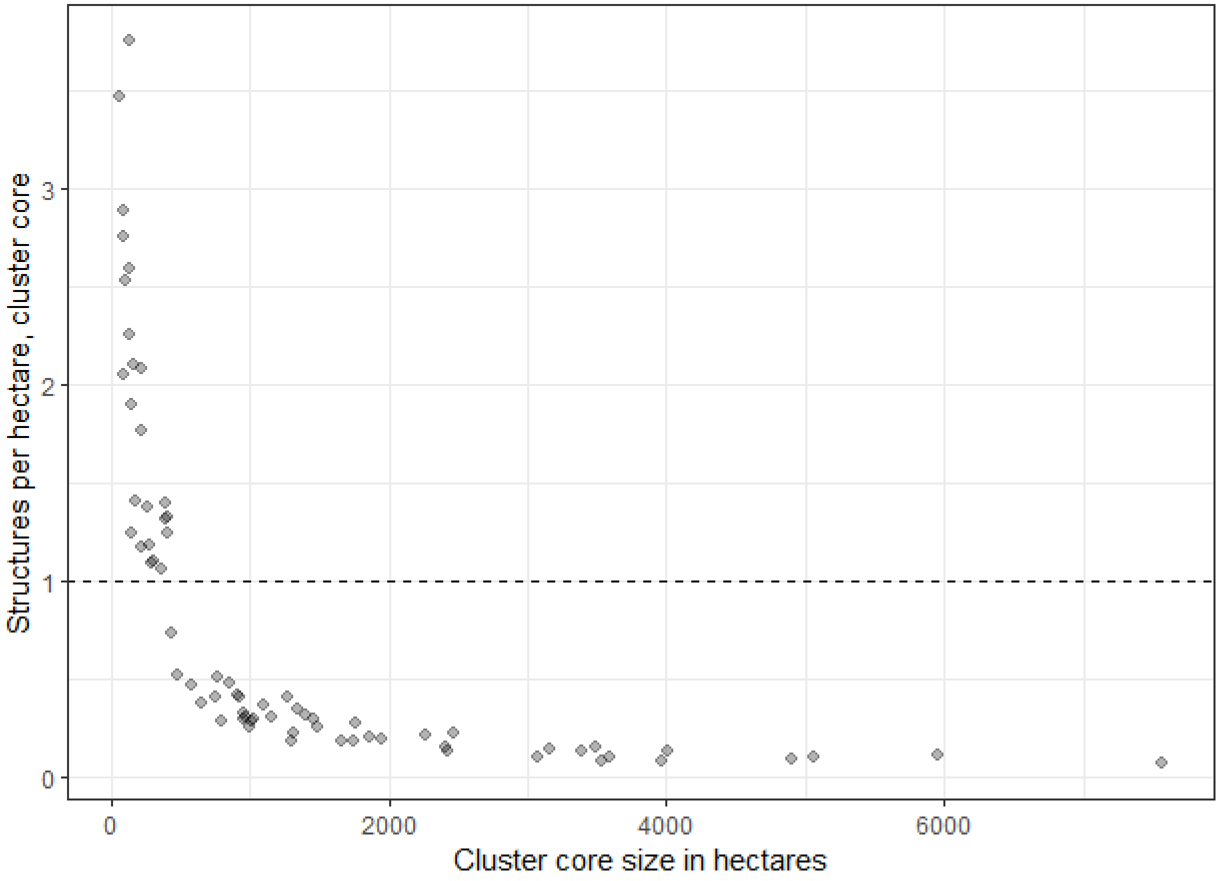

Figure S3: Scatter plot among intervention clusters of ATSB station density versus structure density.
Structure density is defined as total structures enumerated in the pre-trial census in the cluster core divided by cluster core area in hectares. ATSB density is defined as the mean of the total ATSB stations installed in each cluster in the November 2021 and November 2022 installation campaigns prior to the transmissions season, divided by cluster core area in hectares.

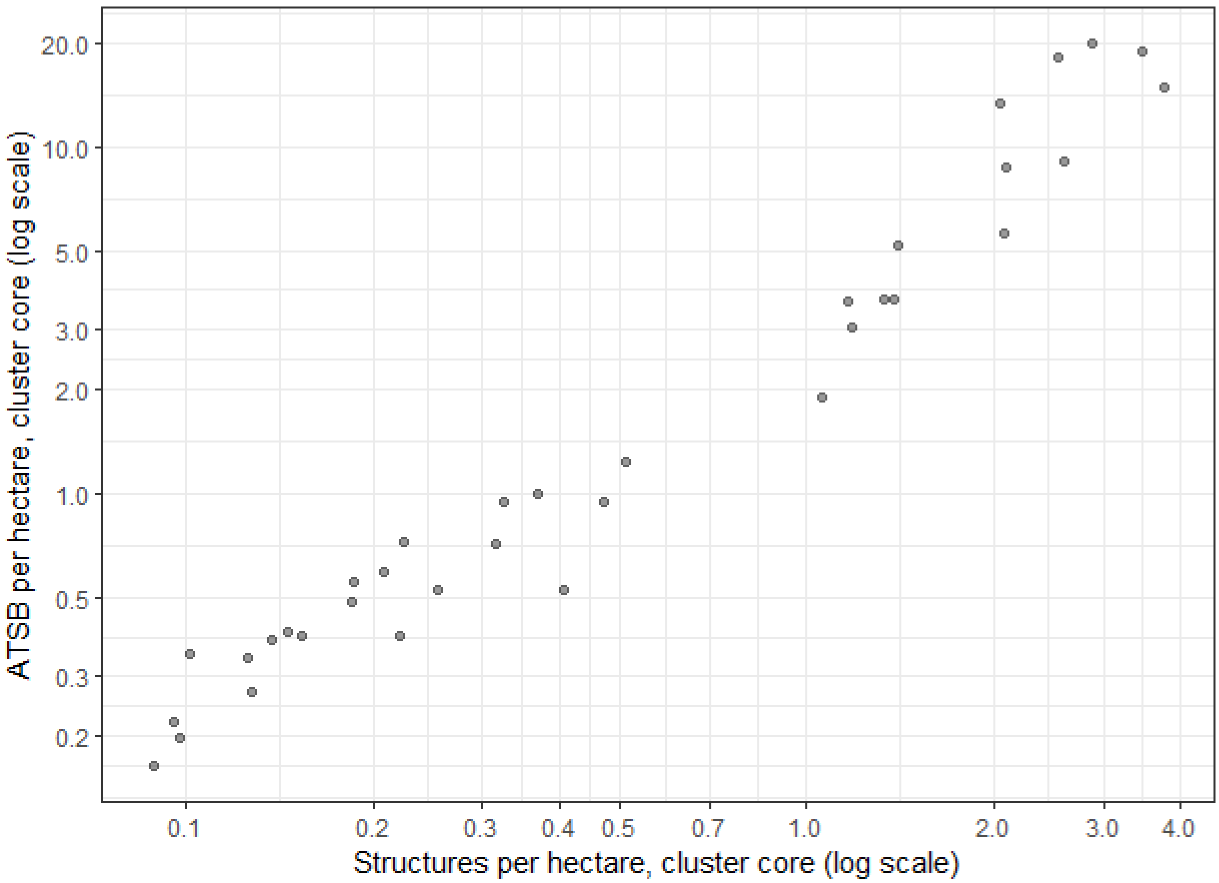

Table S5: Summary of covariate-adjusted ATSB effect estimates on *P. falciparum* infection prevalence

Covariable	ATSB effect, covariate-adjusted model<br>Odds Ratio (OR)	95% CI	p value
Baseline cluster prevalence : low vs. high	0.94	0.72-1.23	0.656
Implementation year	0.89	0.66-1.18	0.415
Age: <5 years vs. 5-14 years vs. 15+ years	0.91	0.67-1.22	0.517
ITN use	0.86	0.64-1.16	0.327
IRS at household	0.88	0.66-1.18	0.406
All restricted randomization covariates	0.89	0.69-1.15	0.367

Table S6: Summary of per-protocol analyses of ATSB effect estimates on *P. falciparum* infection prevalence

Model population	N	ATSB effect, covariate-adjusted model<br>OR	95% CI	p value
Intention-to-treat	2536	0.89	0.66-1.18	0.416
Per-protocol: intervention clusters with ≥80% of eligible structures had ≥2 ATSB in any condition	2465	0.93	0.70-1.24	0.628
Per-protocol: intervention clusters with ≥50% of eligible structures had ≥2 ATSB that did not meet replacement criteria	2355	0.92	0.69-1.22	0.558

Table S7: Summary of adverse events by trial arm

Denominator refers to the total number of child-visits over the cohort follow-up period.

Symptom	Overall<br>Child-visits	n (%)	ATSB arm<br>Child-visits	n (%)	Control arm<br>Child-visits	n (%)
Body swelling	25,858	146 (0.6)	12,942	80 (0.6)	12,916	66 (0.5)
Cough	25,850	7538 (29)	12,938	3755 (29)	12,912	3783 (29)
Diarrhoea	25,847	1448 (5.6)	12,935	743 (5.7)	12,912	705 (5.5)
Dizziness	25,537	853 (3.3)	12,766	442 (3.5)	12,771	411 (3.2)
Eye irritation	25,811	643 (2.5)	12,917	350 (2.7)	12,894	293 (2.3)
Facial oedema	25,859	157 (0.6)	12,943	84 (0.6)	12,916	73 (0.6)
Headache	25,711	8806 (34)	12,869	4364 (34)	12,842	4442 (35)
Itching	25,854	1325 (5.1)	12,941	706 (5.5)	12,913	619 (4.8)
Rash	25,856	1939 (7.5)	12,941	1044 (8.1)	12,915	895 (6.9)
Skin blister	25,856	607 (2.3)	12,942	284 (2.2)	12,914	323 (2.5)
Sore mouth or nose	25,859	497 (1.9)	12,943	261 (2.0)	12,916	236 (1.8)
Vomiting	25,849	1946 (7.5)	12,938	967 (7.5)	12,911	979 (7.6)
Other symptom	25,845	1220 (4.7)	12,935	611 (4.7)	12,910	609 (4.7)

Table S8: Summary of adverse events between children who received artemether-lumefantrine on the previous visit and those who did not receive treatment
Denominator refers to the total number of child-visits over the cohort follow-up period.

	**Overall**		**Did not receive AL on previous visit**		**Received AL on previous visit**	
**Symptom**	**Child-visits**	**n (%)**	**Child-visits**	**n (%)**	**Child-visits**	**n (%)**
Body swelling	25,858	146 (0.6)	21,441	128 (0.6)	4,417	18 (0.4)
Cough	25,850	7538 (29)	21,434	6153 (29)	4,416	1385 (31)
Diarrhoea	25,847	1448 (5.6)	21,432	1202 (5.6)	4,415	246 (5.6)
Dizziness	25,537	853 (3.3)	21,188	670 (3.2)	4,349	183 (4.2)
Eye irritation	25,811	643 (2.5)	21,403	537 (2.5)	4,408	106 (2.4)
Facial oedema	25,859	157 (0.6)	21,442	131 (0.6)	4,417	26 (0.6)
Headache	25,711	8806 (34)	21,317	7095 (33)	4,394	1711 (39)
Itching	25,854	1325 (5.1)	21,437	1077 (5.0)	4,417	248 (5.6)
Rash	25,856	1939 (7.5)	21,440	1606 (7.5)	4,416	333 (7.5)
Skin blister	25,856	607 (2.3)	21,440	505 (2.4)	4,416	102 (2.3)
Sore mouth or nose	25,859	497 (1.9)	21,442	399 (1.9)	4,417	98 (2.2)
Vomiting	25,849	1946 (7.5)	21,434	1562 (7.3)	4,415	384 (8.7)
Other symptom	25,845	1220 (4.7)	21,429	1003 (4.7)	4,416	217 (4.9)
